# Pretreatment Hemoglobin Level Is an Independent Prognostic Factor in Patients with Lung Adenocarcinoma

**DOI:** 10.1155/2018/6328127

**Published:** 2018-05-15

**Authors:** Yue-Hua Zhang, Yuquan Lu, Hong Lu, Meng-Wei Zhang, Yue-Min Zhou, Xiang-Lei Li

**Affiliations:** ^1^Department of Oncology, Huaihe Hospital of Henan University, Kaifeng 475001, China; ^2^Department of Oncology, The First Affiliated Hospital of Henan University, Kaifeng 475001, China; ^3^International Joint Research Laboratory for Cell Medical Engineering of Henan, Huaihe Hospital of Henan University, Kaifeng 475001, China; ^4^Center of Translational Medicine Center, Huaihe Hospital of Henan University, Kaifeng 475001, China

## Abstract

**Background/Aim:**

Few studies have reported the prognostic value of pretreatment hemoglobin levels in patients with lung adenocarcinoma (LA). In the present study, we retrospectively reviewed 306 LA patients for their prognosis associated with the pretreatment hemoglobin levels.

**Methods:**

Person-years and case fatality rate (CFR) were calculated from May 2010 to June 2017. Hazard ratio (HR) and 95% confidence intervals (CIs) were estimated using the Cox proportional hazards regression analysis. Survival curves were generated using the Kaplan–Meier analysis.

**Results:**

Patients with low pretreatment hemoglobin (LPHb) levels had a higher CFR than did patients with normal pretreatment hemoglobin (NPHb) levels (HR = 1.48, 95% CI = 1.06–2.08, and *P*=0.023). Overall survival of NPHb patients was significantly higher than that of LPHb patients (*P* < 0.05).

**Conclusion:**

Low pretreatment hemoglobin level was demonstrated to be an independent biomarker for poor prognosis in patients with LA.

## 1. Introduction

Lung cancer is a common cause of mortality for both men and women [1]. Despite advances in treatment, the five-year overall survival (OS) rate is only 16.3% [2]. In the majority of cases, >80% of lung cancer diagnoses are of the non-small cell lung cancer (NSCLC) type [3], and there are more and more evidences to associate low level of pretreatment hemoglobin with poor survival in patients with NSCLC [4–6]; however, there was no report to associate pretreatment hemoglobin level with the prognosis of adenocarcinoma, a major subtype of NSCLC.

In the present study, we aim to investigate the prognostic value of pretreatment hemoglobin levels for the survival of patients with LA.

## 2. Materials and Methods

From May 2010 to June 2017, 736 patients with lung cancer were diagnosed at the Henan University Huaihe Hospital (Henan, China). The clinical data were retrospectively collected. After excluding 430 ineligible subjects, a total of 306 patients with LA (152 men and 154 women) were selected as subjects for the present study. All cases of LA were pathologically confirmed. The survival period for each subject was defined as the number of days from the date of diagnosis to the date of mortality. Person-years were calculated for each subject. Patients were included in this study if they had a verified diagnosis of LA, regardless of whether they had received prior lung lobectomy, chemotherapy, or radiotherapy treatments.

The clinical stage was assigned on the basis of the 8th Edition of the TNM Classification for Lung Cancer [7]. Anonymous data regarding age, sex, histological cancer type, TNM stage, Karnofsky performance status (KPS) [8], lung lobectomy, chemotherapy, radiotherapy, smoking status, alcohol consumption, family history, diagnosis date, hemoglobin levels, and date of mortality were obtained retrospectively from the patients' medical records, local death registration departments and telephone follow-ups. The study was approved by the Medical Ethics Committee of the Henan University Huaihe Hospital.

The pretreatment hemoglobin levels of the patients were obtained. The LPHb level was defined as <120 g/l of hemoglobin in men, and as <110 g/l in women. All patients were dichotomized into an LPHb group (*n*=53) and a normal pretreatment hemoglobin (NPHb) group (*n*=253). Comparisons of clinical characteristics between the LPHb and NPHb groups were conducted using the chi-squared (*χ* ^2^) test. For univariate Cox proportional hazards regression, age, sex, TNM stages, KPS scores, lung lobectomy status, chemotherapy, radiotherapy, smoking status, alcohol consumption, family history, and hemoglobin levels were dichotomized into a favorable group and an unfavorable group. Hazards ratios (HRs) and 95% confidence intervals (CIs) were calculated to estimate associations between the observed factors and case fatality rate (CFR) of patients with LA. A subsequent multivariate analysis using the Cox proportional hazards model estimated the prognostic influence of age, sex, TNM stage, KPS, lung lobectomy, chemotherapy, radiotherapy, and hemoglobin levels on the CFR of patients with LA.

Survival curves were generated using the Kaplan–Meier analysis method, and the log-rank test was used to examine differences in survival between the various hemoglobin groups. All statistical analyses were performed using Stata software version 13 (Stata Corporation, College Station, TX, USA). *P* < 0.05 was considered statistically significant for all analyses.

## 3. Results

As shown in Table 1, of the 306 patients, 118 (38.6%) were smokers and 38 (12.4%) were alcohol drinkers. In total, 172 (56.2%) patients were at TNM stage IV and 134 (43.8%) were at stage I–III including those unidentified.

Statistical analysis showed that there were significant differences in hemoglobin levels between men and women (*P*=0.020), KPS scores (*P*=0.003), smokers and non-smokers (*P*=0.008), alcohol drinkers and non-drinkers (*P*=0.043), and survival time (*P*=0.005).

The univariate Cox proportional hazards regression analysis showed that patients who were older than 65 years of age (HR = 1.37 and 95% CI = 1.05–1.78), were at TNM stage IV (HR = 2.16 and 95% CI = 1.65–2.82), had KPS scores < 80 (HR = 1.88 and 95% CI = 1.45–2.43), had not received lung lobectomy (HR = 2.78 and 95% CI = 2.02–3.83), had not received chemotherapy (HR = 1.48 and 95% CI = 1.14–1.91), drank alcohol (HR = 1.69 and 95% CI = 1.17–2.45), or had LPHb levels (HR = 1.61 and 95% CI = 1.16–2.24) had a significantly increased CFR. However, sex, radiotherapy, smoking status, and family history did not have any significant associations with the CFR of patients with LA (Table 2).

The multivariate Cox proportional hazards regression analysis showed that LPHb levels were independently associated with an increased CFR (HR = 1.48 and 95% CI = 1.06–2.08). In addition, lung lobectomy (HR = 2.01, 95% CI = 1.32–3.05), chemotherapy (HR = 1.47 and 95% CI = 1.10–1.95), and alcohol drinking (HR = 1.60, 95% CI = 1.06–2.41) were also independent and favorable prognostic factors (Table 3).

**Figure 1:**
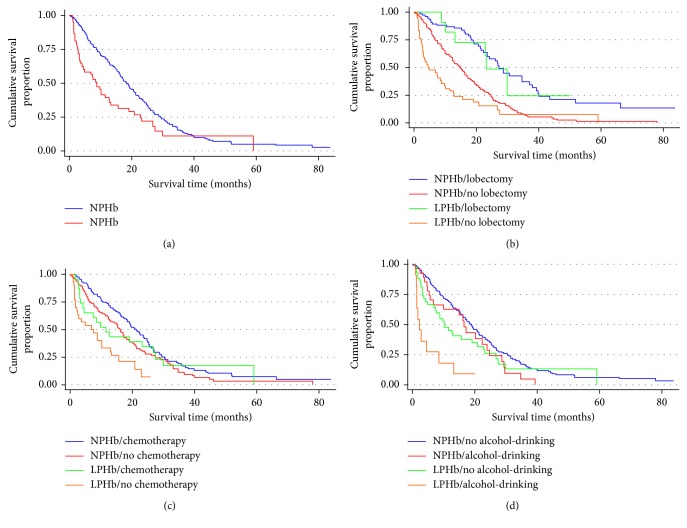
Cumulative survival proportion of patients with LA according to their pretreatment hemoglobin levels. Normal pretreatment hemoglobin (NPHb): men 120–160 g/l, women 110–150 g/l; low pretreatment hemoglobin (LPHb): men < 120 g/l and women ≤ 110 g/l). (a) Overall survival (OS) of NPHb and LPHb patients with LA (log-rank test, *χ* ^2^ = 8.28, and *P*=0.004). (b) LPHb patients had a poorer OS than NPHb patients in the no-lung lobectomy group (log-rank test, *χ* ^2^ = 7.69, *P*=0.006) but not among the lung lobectomy group (log-rank test, *χ* ^2^ = 0.13, *P*=0.7154). (c) The LPHb group had a poorer OS than NPHb patients in the no-chemotherapy group (log-rank test, *χ* ^2^ = 12.00, and *P* < 0.001) but not among the chemotherapy group (log-rank test, *χ* ^2^ = 0.63, and *P*=0.427). (d) LPHb patients had a poorer OS than NPHb patients among alcohol drinkers (log-rank test, *χ* ^2^ = 12.14, and *P* < 0.001), but not among alcohol non-drinkers (log-rank test, *χ* ^2^ = 3.22, and *P*=0.073).

Kaplan–Meier survival curve estimations showed that patients with LPHb had a poorer OS than did patients with NPHb levels (log-rank test, *χ* ^2^ = 8.28, and *P*=0.004, Figure 1(a)). When the patients were subdivided by lung lobectomy, the LPHb patients had a poorer OS than the NPHb patients in the no-lung lobectomy group (log-rank test, *χ* ^2^ = 7.69, and *P*=0.006, Figure 1(b)), a difference not observed in the lung lobectomy group (log-rank test, *χ* ^2^ = 0.13, and *P*=0.7154, Figure 1(b)).

When we subdivided the patients according to chemotherapy, the LPHb group had a poorer OS than the NPHb patients in the no-chemotherapy group (log-rank test, *χ* ^2^ = 12.00, and *P* < 0.001, Figure 1(c)) but not in the chemotherapy group (log-rank test, *χ* ^2^ = 0.63, and *P*=0.427, Figure 1(c)). The subdivision of patients according to alcohol drinking also showed that the LPHb patients had a poorer OS than the NPHb patients among alcohol drinkers (log-rank test, *χ* ^2^ = 12.14, and *P* < 0.001, Figure 1(d)), but not among alcohol non-drinkers (log-rank test, *χ* ^2^ = 3.22, and *P*=0.073, Figure 1(d)).

## 4. Discussion

Our data suggest that pretreatment hemoglobin level, measured at the time of diagnosis, may be an independent predictor for the prognosis of patients with LA, as is the case in the no-lung lobectomy and/or no-chemotherapy patients. To the best of our knowledge, this is the first report that associated pretreatment hemoglobin levels with the prognosis of patients with LA.

Low hemoglobin is common in oncological diseases, such as lung [9, 10], breast [10], gastric [11], and ovarian cancer [12]. There is evidence for a correlation between hemoglobin levels and the prognosis of patients with NSCLC. But precise underlying mechanisms are not fully understood. Tumor cells secrete a number of soluble molecules, including interleukin-6 (IL-6) and tumor necrosis factor-*α* (TNF-*α*). These molecules could decrease hemoglobin by changing the hematopoietic environment [13, 14], suppressing erythropoiesis and erythropoietin (EPO) [15], and impairing the EPO response in erythroid progenitor cells [16]. Moreover, in patients with bone metastasis, bone marrow involvement may lead to bone morrow failure, which may then cause low-hemoglobin levels [17] and subsequently lead to hypoxia, which could induce genomic changes and enhance the development of malignancy [18]. Hypoxia may also boost tumor angiogenesis and accelerate metastasis [19]. Moreover, hypoxia may enhance tumor cell resistance to chemotherapy and radiotherapy through the development of multidrug resistance [20].

For associations of hemoglobin level with lung adenocarcinoma, we did not find previous studies and know little about the mechanisms beneath the surface of water. There was no difference in OS for lung lobectomy group based on Hb, and similar result was found in the chemotherapy group. This is not in line with the previous study of LPHb in patients with non-small cell lung cancer [21]. In a previous study of lung adenocarcinoma patients with LPHb, because of lack of data, we could not analyze the reason. In the present study, about half of the patients abandoned clinical treatment (Supplementary Table 1), and their OS was significantly shorter than those who had received clinical treatments (Supplementary Table 2). We thus suggest that it is difficult to exclude possibilities that it is the clinical treatment that had obscured associations of pretreatment hemoglobin with the final OS. Cigarette smoking was not associated with the OS of LA. This is inconsistent with other studies [22, 23]. We think this maybe related to the fact that we only consider cigarette smoking, without considering the detailed smoking index. But alcohol drinking was significantly and independently associated with the OS of LA, which might be supported by previous studies [24, 25]. Those who had LPHb and drank alcohol had a worst OS.

A major strength of our study was the inclusion of a large number of patients with LA, all with a complete set of clinical data, including the pretreatment hemoglobin levels, the complete survival period, records of multiple treatments, and the family history and lifestyle details, including smoking status and alcohol consumption; this enabled us to investigate the prognostic value of pretreatment hemoglobin levels with decreased sample bias and offset heterogeneity. However, there are also limitations to our study. First, our study was retrospective, and the information on posttreatment recurrence was insufficient. Second, we did not follow alterations of hemoglobin level after pretreatment measurement during clinical treatment and afterwards.

Both lung lobectomy and chemotherapy treatments were associated with the prognosis of patients with LA. However, neither significantly affected the prognostic value of the pretreatment hemoglobin levels in the present study. As for associations of alcohol drinking with the prognosis of LA, we suggest further investigations to confirm the finding.

## 5. Conclusion

Our study suggests that low pretreatment hemoglobin level could be an independent biomarker for poor prognosis in patients with LA. In future clinical studies, the hemoglobin levels should be considered during the work-up of patients with LA in prospective trials, in order to confirm its prognostic significance.

## Figures and Tables

**Table 1:** Pretreatment hemoglobin levels among clinicopathological and lifestyle factors in lung adenocarcinoma patients, Henan, China, 2017.

	NPHb^1^	LPHb^2^	*P* value^3^
Age (years)			
<65	147	24	0.087
≥65	106	29	

Sex			
Man	118	34	0.020
Women	135	19	

TNM^4^ stage			
I–III	115	19	0.200
IV	138	34	

KPS^5^			
≥80	151	20	0.003
<102	33	60	

Lung lobectomy			
Yes	77	11	0.157
No	176	42	

Chemotherapy			
Yes	131	23	0.267
No	122	30	

Radiotherapy			
Yes	36	5	0.351
No	217	48	

Cigarette smoking			
No	164	24	0.008
Yes	89	29	

Alcohol drinking			
No	226	42	0.043
Yes	27	11	

Family history of cancer			
No	234	50	0.636
Yes	19	3	

Survival year			
≥1 year	100	32	0.005
<1 year	153	21	

^1^NPHb (normal pretreatment hemoglobin): men 120–160 g/l and women 110–150 g/l; ^2^LPHb (low pretreatment hemoglobin): men < 120 g/l and women ≤ 110 g/l). ^3^Chi-squared (*χ* ^2^) test. ^4^TNM: tumor-node-metastasis. ^5^KPS: Karnofsky performance status.

**Table 2:** Univariate analysis of prognostic factors for patients with lung adenocarcinoma in Henan, China, 2017.

Factor	Person-years	Cases	Case fatality rate (%)	HR^1^	95% CI^2^	*P* value
Total	421.3	233	55.3			
Age						
<65	263.7	129	48.9	1.00		
≥65	157.7	104	65.9	1.37	1.05–1.78	0.019

Sex						
Man	201.8	120	59.5	1.00		
Women	219.5	113	51.5	0.87	0.67–1.12	0.280

TNM^3^ stage						
I–III	228.9	85	37.1	1.00		
IV	192.5	148	76.9	2.16	1.65–2.82	<0.001

KPS^4^ scores						
≥80	269.5	115	42.7	1.00		
<80	151.8	118	77.7	1.88	1.45–2.43	<0.001

Lung lobectomy						
Yes	172.2	49	28.5	1.00		
No	249.1	184	73.9	2.78	2.02–3.83	<0.001

Chemotherapy						
Yes	248.4	117	47.1	1.00		
No	173.0	116	67.1	1.48	1.14–1.91	0.003

Radiotherapy						
Yes	71.6	32	44.7	1.00		
No	349.7	201	57.5	1.33	0.92–1.94	0.132

Smoking						
No	260.2	138	53.0	1.00		
Yes	161.1	95	59.0	1.09	0.84–1.42	0.500

Alcohol						
No	382.9	200	52.2	1.00		
Yes	38.5	33	85.7	1.69	1.17–2.45	0.006

Family history						
No	385.9	218	56.5	1.00		
Yes	35.4	15	42.4	0.73	0.43–1.24	0.248

Hemoglobin^5^						
NPHb	367.1	189	51.5	1.00		
LPHb	54.3	44	81.0	1.61	1.16–2.24	0.004

^1^HR: hazard ratio by multivariate Cox proportional hazards regression. ^2^CI: confidence interval. ^3^TNM: tumor-node-metastasis. ^4^KPS: Karnofsky performance status. ^5^Hemoglobin: NPHb (normal pretreatment hemoglobin): men 120–160 g/l and women 110–150 g/l; LPHb (low pretreatment hemoglobin): men < 120 g/l and women ≤ 110 g/l.

**Table 3:** Multivariate analysis of prognostic factors for patients with lung adenocarcinoma in Henan, China, 2017.

Factor	HR^1^	95% CI^2^	*P* value
Age			
<65	1.00		
≥65	1.20	0.90–1.60	0.211

Sex			
Man	1.00		
Women	0.89	0.66–1.19	0.432

TNM^3^ stage			
I–III	1.00		
IV	1.39	0.98–1.99	0.067

KPS^4^ scores			
≥80	1.00		
<80	1.24	0.93–1.64	0.139

Lung lobectomy			
Yes	1.00		
No	2.01	1.32–3.05	0.001

Chemotherapy			
Yes	1.00		
No	1.47	1.10–1.95	0.008

Radiotherapy			
Yes	1.00		
No	1.00	0.68–1.49	0.981

Alcohol drinking			
No	1.00		
Yes	1.60	1.06–2.41	0.026

Hemoglobin^5^			
NPHb	1.00		
LPHb	1.48	1.06–2.08	0.023

^1^HR: hazard ratio by multivariate Cox proportional hazards regression. ^2^CI: confidence interval. ^3^TNM: tumor-node-metastasis. ^4^KPS: Karnofsky performance status. ^5^Hemoglobin: NPHb (normal pretreatment hemoglobin): men 120–160 g/l and women 110–150 g/l; LPHb (low pretreatment hemoglobin): men < 120 g/l and women ≤ 110 g/l.

## Supplementary Materials

Supplementary Materials Table 1: distribution of patients with lung adenocarcinoma among TNM stages, chemotherapy, and lung lobectomy. Table 2: treatment modality and survivals (months) of patients with lung adenocarcinoma in Henan, China.
