# Supplementary material for: Pretreatment Hemoglobin Level Is an Independent Prognostic Factor in Patients with Lung Adenocarcinoma
Source: Can Respir J. 2018 May 15;2018:6328127. doi: 10.1155/2018/6328127 (PMC5977049; doi:10.1155/2018/6328127)
Supplement: Supplementary Materials — Table 1: distribution of patients with lung adenocarcinoma among TNM stages, chemotherapy, and lung lobectomy. Table 2: treatment modality and survivals (months) of patients with lung adenocarcinoma in Henan, China. [file 6328127.f1.pdf]

Supplementary Materials

Supplementary Table 1. Distribution of patients with lung adenocarcinoma among TNM stages, chemotherapy and lobectomy.

TNM1 Stage	Lung lobectomy	No. of patients	Chemotherapy		p-value2
			Yes	No	
Total		306	154 (50.3%)	152 (49.7%)	
I-III	Total	134	64 (47.8%)	70 (52.2%)	0.003
	Yes	83	48 (57.8%)	35 (42.2%)	
	No	51	16 (31.4%)	35 (68.6%)	
IV	Total	172	90 (52.3%)	82 (47.7%)	0.575
	Yes	5	2 (40%)	3 (60%)	
	No	167	88 (52.7%)	79 (47.3%)	
p-value			< 0.001		

[1]TNM: tumor-node-metastasis; [2]Pearson Chi-sqare test

Supplementary Table 2. Treatment modality and survivals (months) of patients with lung adenocarcinoma in Henan, china.

Lung lobectomy	Chemotherapy	TMN1 Stage	No. of patients	Survival months2 (mean±Sd)	Min	Max
Yes	Yes	I-III	48	27.1±18.3	3.6	83.8
Yes	Yes	IV	2	22.8±29.6	1.8	43.7
Yes	No	I-III	35	20.1±13.2	2.4	68.3
Yes	No	IV	3	16.5±7.2	10.1	24.3
No	Yes	I-III	16	16.5±10.6	1.3	38.9
No	Yes	IV	88	16.1±12.0	1.7	59.2
No	No	I-III	35	14.9±9.9	1.2	36.5
No	No	IV	79	10.5±12.0	0.1	77.9
Total			306	16.8±14.0	0.1	83.8

[1]TNM: tumor-node-metastasis; [2]Significant difference in suevival months among different treatment modelities (ANOVA test, P<0.001).
